# How COVID-19 pandemic is changing the Africa’s elaborate burial rites, mourning and grieving

**DOI:** 10.11604/pamj.supp.2020.35.23756

**Published:** 2020-06-17

**Authors:** Abidemi Emmanuel Omonisi

**Affiliations:** 1 Department of Anatomic Pathology, Ekiti State University, Ado-Ekiti, Nigeria; 2 Department of Anatomic Pathology, Ekiti State University Teaching Hospital, Ado-Ekiti, Nigeria

**Keywords:** COVID-19 pandemic, burial rites, mourning

## Abstract

There are diverse burial rites in Africa which have been practiced for decades depending on the deceased place of origin, culture, religion and the position held in the community. Unlike the developed countries where burials are usually conducted as private ceremonies, funerals in Africa are elaborate and are usually public ceremonies involving the entire members of families, friends and well-wishers. Religion and culture are usually the deciding factors when decisions are made on how the deceased should be buried but generally cremation is not commonly practiced in Africa. COVID-19 pandemic was generally accepted to originate from Wuhan in China and this pandemic has extended to Africa. Most countries in Africa responded to the COVID-19 pandemic by adopting the same strategies used by the Western countries in curbing the spread of the virus through the imposition of restrictions on movements, lock down and the introduction social distancing rules which are align to Africa way of living. These control strategies had put a lot of pressures on the weak mortuary services in Africa, altered the traditional methods of observing burial rites, mourning and grieving. COVID-19 pandemic has changed the various traditional ways Africans mourn grief and bury their love one. The dead bodies of people suspected or confirmed to have COVID-19 should be treated with respect, ensuring the rights of the dead to a dignifying burial are upheld while adhering to standard precautions including use of appropriate PPEs, hand hygiene before and after the burial procedure.

## Commentary

This is not the first time in history that human coronaviruses will be taking the world by surprised through causing a pandemic disease. In the 21st century, unexpected human coronavirus outbreaks caused Severe Acute Respiratory Syndrome Coronavirus (SARS-CoV) and Middle East Respiratory Syndrome Coronavirus (MERS-Co) in China which were suggested to emerge from bats [1]. Coronavirus disease 2019 (COVID-19) is caused by SARS-COV-2 and is generally believed to emerged from Wuhan in China [2]. This disease has extended to over 206 countries with 1,051,635 confirmed cases, and 56,985 deaths recorded as of 4th April 2020 by World Health Organization (WHO) [3]. The statistics of people infected with coronavirus and dying from COVID-19 keep increasing on a daily basis since the major outbreak of the COVID-19 in many countries of the world. Surprisingly, despite the technological advancement, adequate number of health workers and well developed health systems in the developed countries like United Kingdom, USA and Russia compared with nations in Africa, developed countries had thus far recorded more deaths and were most hit by the COVID-19 pandemic when compared with nations in Africa [4]. Unfortunately, these deaths were not buried conventionally in many countries of the world with the family members of the deceased restricted for performing the usual burial rites and organizing a very “befitting” burial for their deceased members of families from COVID-19 [5,6]. In Ireland, the Irish Association of Funeral Directors advised undertakers not to embalm the deceased, and to hold closed-coffin funerals instead of open-casket events [7]. The pandemic has ended communal prayer and congregational funeral gatherings at many major churches, synagogues, mosques and temples in most countries of the world including Africa with some family members barred from cemeteries. The United Synagogue, a union of Orthodox British Jewish synagogues, postponed the usual stone-setting ceremonies associated with burial in England [8]. All cemeteries have been shut and those sitting shiva, the seven-day period of mourning, have been advised that they cannot have visitors [8].

**The concept of death in Africa:** in Africa, death is regarded as a dreaded event and seen as the beginning of the communication between the visible and the invisible worlds. Some communities in Africa believed that the goal of life is to become an ancestor after death [9]. This is one of the major reasons Africans believed that every person who dies must be given a “befitting” funeral, accomplished by a number of traditional and religious rites and ceremonies as the deceased migrate to join the ancestors in the invisible world [9]. If this is not done, it is generally believed that the dead person may become a wandering ghost and will continue to torment those still living especially members of deceased family who ought to give the deceased a “befitting” funeral. As part of giving a very “befitting” funeral as experienced in Africa, funeral rites are usually incorporated as parts of the fundamental aspects of the funeral ceremonies and in most cases usually precede actual burial of the deceased. The nature and how complex the various funeral rites are usually vary with communities where the deceased originated from, religion of practice, and the background of the dead or position they held in the community before death and their cultures heritage [9].

Funerals in most nations in Africa are organized essentially to mourn and celebrate the life of the deceased [10]. They are very elaborate events, often the central life cycle events, unparalleled in cost and importance, for which families harness vast amounts of resources to host and lavish events for multitudes of people with ramifications well beyond the event [11].The number of people and class of people present during a funeral may be an indication of the character, financial status, position in the community and how sociable the deceased was before death. Characteristically in most nations in Africa, these funeral ceremonies often take place during the weekends; Fridays and Saturdays to ensure the presence of mourners, well-wishers and elaborate events. However, in the Western world, funerals are very private ceremonies, reserved for family and closest friends of the deceased [1].

**Socio-cultural issues, burial rights and dying in Africa:** most Africans do not like thinking, planning, and facing the reality of death. In most nations in Africa, it is a taboo to discuss about one´s own death or the death of loves ones or plan about death. The practice of the living writing wills and budgeting for death is uncommon in Africa. No wonder death is considered by Africans as the last enemy that must be defeated and no matter how hopeless the case might be life must be preserved [9]. In same Africa, some aged parents usually will give verbal instructions to their children concerning how to conduct their burial ceremonies and where to be buried. It is the tradition in most nations in Africa to bury their love one in their ancestral homes where the deceased died [12]. This explains why corpses of Africans who died in the Western countries were flew back and buried in Africa. There are lot of cultural issues and believes attached to where deceased bodies are buried in Africa [12]. It is also one of the prominent cultures in Africa to bring up individual from childhood with a sense of belonging and relatedness with others in a communal fashion. This results to greater bonding with members of his family and community at large. This relationship also plays out at death and during the burial rites as such funerals are usually seen as a communal event when compared to the Western countries where the Individuals are brought up as a member of a nuclear family only.

There are three mains religions in Africa namely; Christianity, Islam and African Traditional religion. These religions have unique burial rites and traditions. The Christians leave the deceased in the morgue for days or weeks even several months, while making preparations for a “befitting” burial. Furthermore, Christianity allows the use of casket for burial and the caskets used for the burial of deceased are usually of different size, shapes, design and sometimes are very unique and colourful. The type of the casket used to bury the deceased may represent the deceased favourite object or profession or favourite object or social status; this could range from an animal shaped casket to a pencil or soft drink bottle, or and aircraft. The quality of the casket may also suggest the financial status of the deceased while alive or the financial status of the family. The rich are usually buried in expensive casket made up of gold or other expensive materials. Some Africans have gone to the extreme by burying their aged parents or deceased rich individuals in an expensive vehicle. The vast majority of the people in Africa are poor and are usually buried using wooden caskets that are very cheap and easily obtainable from trees in the thick forest which abound in Africa.

It is now a common practice in Africa especially, in Nigeria that the beavered families also organize uniforms for the mourners and sympathisers call “asoebi” [13] to make the funeral ceremony very elaborate and colourful when burying their aged parents. In case where a woman loses her spouse, she is expected to dress in black or white to mourn her deceased husband and in some cases shave her entire head hairs during this period as a mark of morning. It is also a common practice that, if a young person dies, everyone attending the funeral is expected to appear in dark colours as a mark of mourning. After the burial the mourners celebrate the life of the deceased through song and dance. After the burial of the aged parents or rich individuals, there is heavy feasting accompanied by singing and dancing to a mix of African rhythms, jazz and brass bands. Many families come back a few years later for the dead celebration called “death remembrance celebration” some sort of second burial or memorial in honour of the dead. They perform rituals and organise celebrations were family members and friends gather to celebrate. This practice is common among the Yoruba tribe of Southwestern, Nigeria. The burial rites for Muslims are complicated, but all of the rituals are intended to respect the body as much as possible, knowing that this person will soon be presented to God. When a Muslim is approaching death, family members and very close friends should be present. They should offer the dying person hope and kindness, and encourage the dying person to say the “shahada” confirming that there is no God but Allah. As soon as death has occurred, those present should say, “Inna lillahi wa inna ilayhi raji’un” (“Verily we belong to Allah, and truly to Him shall we return”) [14].Those present should close the deceased´s eyes and lower jaw, and cover the body with a clean sheet. They should also make “dua” (supplication) to Allah to forgive the sins of the deceased [14].

According to Islamic law (“shariah”), the body should be buried as soon as possible from the time of death, which means that funeral planning and preparations begin immediately. A local Islamic community organization should be contacted as soon as possible, and they will begin to help make arrangements for the funeral service and burial, assist the family in identifying an appropriate funeral home, and coordinate with the funeral home[15]. Embalming and cosmetology are not allowed unless required by state or federal law. Because of the prohibition on embalming and the urgency with which the body must be buried, it is not possible to transport the body from one country to another. To prepare the body for burial, it must be washed (“Ghusl”) and shrouded (“Kafan”). When a Muslim dies, the body should be buried as soon as possible after death, thus there is no viewing before the funeral [15]. Cremation is forbidden for Muslims [16]. Salat al-Janazah (funeral prayers) should be performed by all members of the community [16]. After the funeral and burial, the immediate family will gather and receive visitors. It is customary for the community to provide food for the family for the first few days of the mourning period (usually three days). Generally, the mourning period lasts 40 days, but depending on the degree of religiousness of the family, the mourning period may be much shorter. It is acceptable in Islam to express grief over a death. Crying and weeping at the time of death, at the funeral, and at the burial are all acceptable forms of expression. However, wailing and shrieking, tearing of clothing and breaking of objects, and expressing a lack of faith in Allah are all prohibited [16].

In African traditional religion, generally the dead are buried in their land of ancestry. Burial, to be considered proper, honourable, meaningful and acceptable in most African cultures, has to be done in the deceased´s ancestral land [17]. The native religions of Africa, which is the oldest type of religion in the continent, life does not end with death but continues in another realm and immediately after death,Vmost African traditional believers respect the transitional period of the deceased during which the incorporation of the deceased into the world of the dead and uniting with the ancestors [17]. This is one of the reasons why different kinds of rites and sacrifices will take place during this period. Although, it is impossible to generalize the concepts of burial rites based the African religion because they are diverse ethno-religions group in the continent. Personal belongings are often buried with the deceased in other clans to assist the deceased in the journey. In some communities, when someone has died in a house, all the windows are smeared with ash, all pictures in the house turned around and all mirrors and televisions and any other reflective objects covered. The beds are removed from the deceased’s room, and the bereaved women sit on the floor, usually on a mattress. Most Africans believed that deaths are not natural but a spiritual event [18]. During the time preceding the funeral usually from seven to thirteen days visits are paid by people in the community to comfort the bereaved family and some sacrifices are also performed during this period [19]. The African religion basically adapted the ancient customs in the continent for funerals and the bottom line is that many African burial rites begin with the sending away of the departed with a request that they do not bring trouble to the living, and they end with a plea for the protection of those alive by their ancestors [20].

**Coronavirus changes the burial rites, mourning and grieving in Africa:** to curb the ravaging menace of coronavirus, several governments across Africa are currently on a lockdown with various laws instituted that forbid mass gathering of more than 20 people in a location at same time including banning of burial and funeral ceremonies, weddings parties, religious gatherings , imposition of restriction of movement and introduction of social distancing [21]. In Africa, the mortuaries pre- COVID-19 pandemic era are usually crowded from Thursdays as deceased relatives visit the mortuaries to make all necessary preparations to pick their deceased ones for burials usually scheduled for Fridays or Saturdays in most countries in the continent. The ban on mass gathering and funeral during this period of lockdown significantly reduced the patronage and significant reduction in the number of corpses released to the beavered families in most mortuaries. Bodies are now pilling up in most state mortuaries across the continent [22]. The mortuaries are now shadow of themselves with the mortuary attendants doing less of releasing bodies and the funeral directors now practically on holidays due to the marked reduction in burial activities across the nations in Africa. Few family members that visited the mortuaries are not allowed to touch or kiss dead bodies as part of the precautionary measures now in place to combat the on-going COVID-19 pandemic, a practice that is very common in some cultures in Africa. The relatives and friends of the deceased are not allowed to visit the mortuary in large numbers during this COVID-19 pandemic. Only few of them usually less than 20 people are allowed to mourn and grief with deceased at the mortuaries. They are also asked to use face masks and also observe the social distancing and other rules associated in curbing COVID-19.

Burial ceremonies are now less elaborate events in Africa because of the COVID-19 pandemic. Most Africans that has course to bury their relatives did that without or in few cases with very minimal burial rites done during this period of pandemic. The usual elaborate ceremonies with associated feastings are lacking in most burial conducted during this COVID-19 pandemic. These are unusual ways of mourning and burying deceased individuals in Africa. Despite the ban on burial and funeral ceremonies, human beings continue to die both from COVID-19 pandemic and other causes of deaths, leading to accumulation of corpses in the mortuaries. Unfortunately, a proactive communication strategy was not put in place to educate and inform the population on the rationale behind rapid burial, or burial in an exceptional fashion of corpses from COVID-19. It is important to mention that, most mortuaries in Africa have a small capacity to store bodies and are poorly equipped with instruments, and lack adequate trained human resources to cope with mass deaths during pandemics like COVID-19 [23]. With the sustained lockdown, most mortuaries are filled to the brim and corpses are laid on the floor in some mortuaries in Africa. This is not the best time for families who have lost their love ones as the rejection of corpses are now a common practice in most mortuaries in Africa. The rejection of some corpses due to lack of space in the mortuaries to store the corpses has been forcing the relatives to bury their ones immediately and postponing the burial ceremonies until the COVID-19 pandemic will be over. Expectedly in Africa, like what was experienced during the Ebola outbreak [24], some mortuaries have stopped receiving bodies especially those from outside their territories because of fear of contacting COVID-19. This development had also compelled relatives to bury their deceased love ones immediately because of the widespread rejection and scrutiny of dead bodies in most mortuaries.

There are some fears whether coronavirus is still active after an individual has died. Although, World Health Organization (WHO) has categorically stated that till date, there is no evidence of persons having become infected from exposure to the bodies of persons who died from COVID-19 [25]. The fear of possible spread from COVID-19 dead bodies to the living has led to the abandonment of some burial rites such as bathing particularly those that died from coronavirus. Body bags are now used to replace the kafan, or white burial shroud by the Muslims and the routine washing of the body procedure changed to another Islamic method, tayammum, which consists of cleaning the dead body with either sand or dust [26]. Unfortunately, some COVID-19 infected Muslims who died from the disease have their bodies cremated against the Islamic the Islamic burial rites which forbid cremation of body despite rejection of such procedure by Muslims community [27]. It is important to mention that, some Muslims in some countries still observe all the Muslim burial rituals including the washing of the dead bodies but those involved in the burial procedure worn personal protective equipments to prevent possible transmission of the virus [28]. COVID-19 pandemic has also caused the bodies of most immigrants not to be laid to rest in their countries of origin because of the suspension of international flights and restriction of movements. The COVID-19 dead body were buried in most countries and cities where they died. The practice by some cultures in burying their love ones in their places of origin is now hard to fulfil during this pandemic. All these have brought several changes to the way Africans mourn and conduct burial rites irrespective of religious believe and culture [29].

## Conclusion

COVID-19 pandemic has changed the various traditional ways Africans mourn grief and bury their love one. The traditional burial rites that may necessitate direct contact with dead bodies are now being abandoned. The various laws enacted and implemented in the various nations in Africa have prevented mass gatherings for morning, praying for the dead bodies and celebration of the deceased through feasting. There is need for policy makers and health care providers usually members of the burial teams to meet with the families before the burial of corpses of COVID-19. This meeting will serve as an avenue to address all the various concerns by the relatives on how and why the corpses of COVID-19 are handled in line with the protocol on handling of such corpses. This will also enable the families to be in agreement with the burial team and policy makers on the proper way of disposing such bodies in line with the appropriate protocol. However, despite the COVID-19 pandemic, dead bodies of people suspected or confirmed to have COVID-19 should be accorded respectful burial through consulting with the deceased families in ensuring the deceased culture, belief system and religion are observed and the bodies are properly handled in a dignifying manner before the burial. It is advocated that adhering to standard precautions including use of appropriate PPEs, hand hygiene before and after the burial procedure should be properly adhered to all through the funeral.

## Competing interests

The authors declare no competing interests.
